# Towards portable MRI in the plant sciences

**DOI:** 10.1186/s13007-024-01152-z

**Published:** 2024-02-18

**Authors:** Shannan Blystone, Magali Nuixe, Amidou Sissou Traoré, Hervé Cochard, Catherine Picon-Cochard, Guilhem Pagés

**Affiliations:** 1 Université Clermont Auvergne, INRAE, UR QuaPA, 63122 Saint-Genès-Champanelle, France; 2 INRAE, PROBE research infrastructure, AgroResonance facility, 63122 Saint-Genès-Champanelle, France; 3 Université Clermont Auvergne, INRAE, PIAF, 63000 Clermont-Ferrand, France; 4 Université Clermont Auvergne, INRAE, VetAgro Sup, UREP, 63000 Clermont-Ferrand, France

**Keywords:** Cavitation, Flow, Low-field NMR, Plant physiology, Relaxation times, Structure, Water

## Abstract

Plant physiology and structure are constantly changing according to internal and external factors. The study of plant water dynamics can give information on these changes, as they are linked to numerous plant functions. Currently, most of the methods used to study plant water dynamics are either invasive, destructive, or not easily accessible. Portable magnetic resonance imaging (MRI) is a field undergoing rapid expansion and which presents substantial advantages in the plant sciences. MRI permits the non-invasive study of plant water content, flow, structure, stress response, and other physiological processes, as a multitude of information can be obtained using the method, and portable devices make it possible to take these measurements in situ, in a plant’s natural environment. In this work, we review the use of such devices applied to plants in climate chambers, greenhouses or in their natural environments. We also compare the use of portable MRI to other methods to obtain the same information and outline its advantages and disadvantages.

## Introduction

Plant physiology and structure are constantly changing according to phenology, environmental conditions, or exposure to biotic or abiotic stress. The study of plant water dynamics can give information on these changes, as they are linked to numerous plant functions. Photosynthesis, the fixation of atmospheric carbon, which is requisite to plant growth, implies heavy water consumption. For every mole of CO_2_ sequestered as a result of photosynthesis, around 200 mol of water are lost to transpiration through stomata that are open for gas exchange [1]. Thus, a plant must balance its need for CO_2_ with the water loss that necessarily occurs with its uptake. To fulfil this need, water travels through a soil-plant-atmosphere continuum, where it is absorbed from the soil by the roots, and then is transferred to other plant organs through the xylem. The xylem carries water to the leaves, where photosynthesis uses water as a reactant and produces carbon-rich sugars, then it enters the atmosphere as water vapor through the stomata. Meanwhile, the carbon-rich sugars are transported by the phloem and distributed to the plant’s organs and carbon sinks. The coupling of the water and carbon cycles is at the foundation of plant ecological and physiological functioning and renders the understanding of plant water dynamics vital.

One of the difficulties in the plant sciences is being capable of measuring plant water dynamics non-invasively and in a plant’s natural environment, i.e., in situ. Nuclear magnetic resonance (NMR), and its imaging counterpart, magnetic resonance imaging (MRI), provide a suitable way to non-invasively assess plant water status throughout the plant’s life cycle, and portable devices offer the possibility of taking measurements in a plant’s natural environment. Recently, reviews have been published addressing the use of this technique in the agricultural and food sciences [2, 3]. Herein, we present the utility of studying water in plants with portable MRI by looking at the different parameters that can be measured with the method, such as plant water content, xylem and phloem flow, plant structure, physiological changes, and disease progression. We then compare the use of portable MRI to other available methods to measure the same parameters. We use the term “portable MRI” to refer to any device that can or could be transported into the field and the term “laboratory MRI” to refer to any device that cannot be transported. This review is limited to the use of portable or potentially portable devices applied to the scale of individual, intact plants in climate chambers, greenhouses, or outdoors, and to the study of detached plant organs offering physiological insight into whole-plant processes.

### Overview of NMR/MRI

MRI is the analytical technique of choice to study water and its properties without ionizing or degrading the object. In this section, we will present a brief overview of the basics of NMR and MRI. As we will not explain the physical principles, the interested reader can refer to any textbook.

Hydrogen nuclear magnetic resonance is based upon the magnetic properties of hydrogen nuclei, also called protons. When placed inside a magnetic field (B_0_) and excited by a radiofrequency pulse at the specific nucleus resonance frequency, an NMR signal can be detected. This signal can be described as a decay (Eq. 1) characterized by two relaxation times, the longitudinal relaxation time (*T*_1_) and the transverse relaxation time (*T*_2_).

1 $$S\left( t \right) \propto \left( {1 - { }exp^{{{\raise0.7ex\hbox{${ - t}$} \!\mathord{\left/ {\vphantom {{ - t} {T_{1} }}}\right.\kern-0pt} \!\lower0.7ex\hbox{${T_{1} }$}}}} } \right)\left( {exp^{{{\raise0.7ex\hbox{${ - t}$} \!\mathord{\left/ {\vphantom {{ - t} {T_{2} }}}\right.\kern-0pt} \!\lower0.7ex\hbox{${T_{2} }$}}}} } \right)$$

Longitudinal relaxation corresponds to the return of the magnetization to its equilibrium state in the longitudinal plane, which is the direction of B_0_. *T*_1_ is the time it takes to recover 63% of the initial magnetization along the longitudinal axis. Transverse relaxation corresponds to the dephasing of the spins and depends on the interactions of the spins with their environment. *T*_2_ is the time required for the NMR signal to decrease by 63% of its initial value in the transverse plane. Water molecules bound to macromolecules or surfaces will exhibit shorter relaxation times than more freely moving water molecules. As measured relaxation times also depend on the magnetic field, care should be taken when comparing studies, and only the order of magnitude of the values should be compared. Relaxation times measured in plants generally range from a few milliseconds to a few hundred milliseconds.

**Fig. 1:**
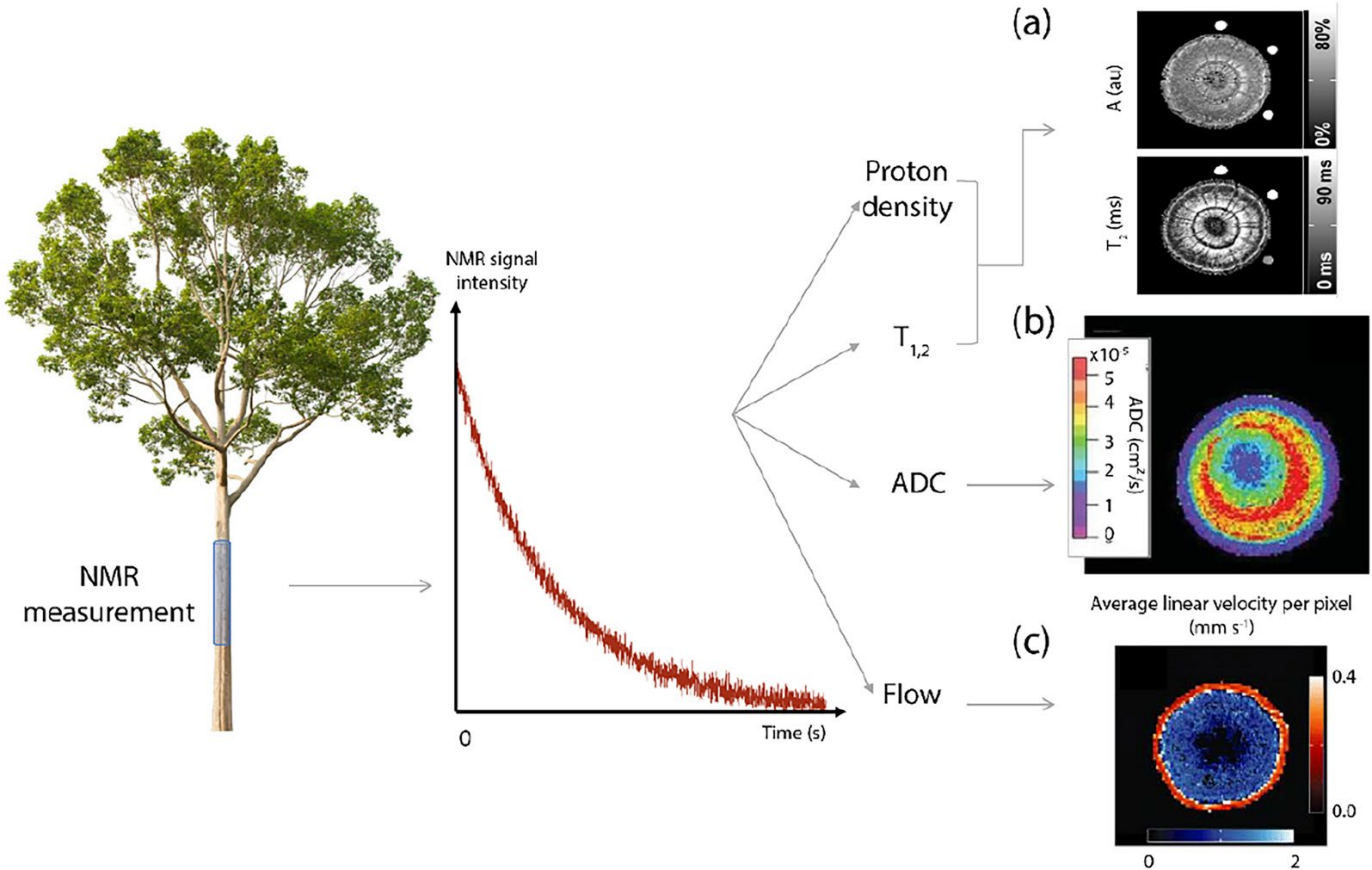
According to the weighting of the NMR decay, different parameters can be measured such as proton density, longitudinal (*T*_1_) and transverse (*T*_2_) relaxation times, diffusion (ADC) and flux. Subset (a) represents proton density and *T*_2_ maps acquired on a *Fagus sylvatica* stem (Adapted from [10] with permission from Wiley). Subset (b) represents an ADC map acquired on a pear tree (Adapted from [11] with permission from AIP Publishing). Subset (c) represents an example of flux measurements. It corresponds to the average linear velocity map of a poplar tree stem (Adapted from [12] with permission from Blackwell Publishing Ltd). The red scale corresponds to the velocity measured in the phloem, while the blue scale corresponds to the velocity measured in the xylem

Magnetic resonance imaging allows the imaging of water by adding spatial information. The NMR signal is directly proportional to the quantity of water within the measured zone, making it possible to obtain an image of the distribution of the water concentration (also called proton density) in the sample. Magnetic resonance techniques also permit the analysis of the interaction of water with tissues. Using magnetic resonance, it is possible to obtain relaxation time spectra, to create images weighted by these relaxation parameters, or to obtain quantitative images of these relaxation parameters, i.e., the value of a given parameter (e.g., *T*_2_) is assigned to each voxel of an image. Such parametric images permit the study of organ or cell structure [4, 5]. Magnetic resonance techniques also offer the possibility of studying water dynamics. To do so, protons are spatially labelled (non-invasively by using a pulsed field gradient) and either the apparent diffusion coefficient (ADC) or the probability of the average velocity of flowing water can be measured (Fig. 1). Thus, by studying the water within a plant through a variety of different magnetic resonance approaches, one can gain a multitude of information, not just on water content, but also on plant structure or physiological processes, such as stress response or the progression of a disease. For example, laboratory MRI has long been used to measure the water dynamics in plants, follow growth, or understand plant structure [6–9]. The main advantage of laboratory MRI is that the stronger magnetic fields make it more sensitive, yielding a better signal-to-noise ratio (SNR). However, these instruments are expensive to maintain and are not portable. The incapacity to measure plants in situ has rendered laboratory MRI a tool of limited use in the plant sciences as the environment of a plant is primordial to its physiological functioning.

To measure plants in their natural environment, a portable device must be used. Such instruments exist but are mostly made in-house. As lightweight magnets must be used, the magnetic field strengths of portable devices are generally lower than 1 T, commonly referred to as low-field devices. The spatial resolution of such devices can range from ~ 100 μm [10, 12] to a couple of millimeters [13], while the temporal resolution can range from minutes to a few hours, depending upon the experiment. The balance between spatial and temporal resolution depends largely on the SNR, which is lower in portable devices due to the lower magnetic field strength. Another challenge with portable devices, beyond reducing magnet size, is optimizing magnet shape for a variety of samples. C-shaped, U-shaped and Halbach magnets enclose the magnetic field, offering field homogeneity, and Halbach magnets in particular offer the highest field strength per mass of material. However, all these magnets are limited by their internal diameter and accessibility. Unilateral magnet designs have the advantage of being able to accommodate samples of any size and shape, however, the measurement depth is limited so that only a small part of a larger sample can be measured. Magnet choice is thus a compromise between the desired resolution, portability, and accessibility, and an ideal magnet will depend upon the experiment and the sample. Portable magnets have been developed that are suitable for outdoor environments, and previous works have shown that portable MRI stands as an interesting analytical tool to investigate water status and movement in plants in situ. In this review, we will discuss some of these works and show how studying water in plants with MRI can give information on plant water content, and also xylem and phloem fluxes, anatomy, growth, stress response, disease, and other physiological processes. We will then compare portable MRI to alternative methods to obtain the same information.

### Overview of methods to study water in plants alternative to MRI

In this section, we will briefly review some of the most commonly used methods to measure water content, plant structure, xylem and phloem flow, and cavitation. We consider a method as non-invasive when it does not entail equipment entering the sample body or otherwise damaging the plant. We consider a method as destructive when the sample is destroyed after use of the method and no longer viable for future measurements.

Water content can be measured by taking fresh and dry weights of plant matter [14]. From the fresh and dry weights and the inclusion of turgid weight as a variable, one can directly estimate a plant’s relative water content (RWC). This method normalizes data to account for differences in morphology between plants. Water content can also be estimated using near infrared spectroscopy (NIRS). NIRS is based upon the absorption of NIR light by a sample. Light is absorbed in a wavelength region depending upon the composition of the sample, and this permits the acquisition of a spectrum with peaks indicating the quantity of certain components, such as water [15]. Terahertz (THz) spectroscopy is also capable of measuring water content non-invasively, and functions by applying pulses of THz radiation to the sample and analyzing its transmission. As water is a strong absorber of THz waves, the THz spectrum correlates to the quantity of water in the sample [16].

To study plant structure, standard methods include dye tracing, microscopy techniques, X-ray tomography [17, 18], neutron radiography [19–21], air-coupled ultrasound [22], laser-based guided waves [23], and 2D-light transmission [24, 25]. Dye tracing has been used to study plant structure for almost a century [26], and functions by staining certain anatomical structures, and the distribution of dyes can be observed either macroscopically or under the microscope. Microscopy techniques are also well established and can be used to study microstructures within a certain depth. X-ray radiography and tomography rely upon the attenuation of X-rays passing through the sample, which is rotated throughout the measurement process, permitting the reconstruction of 2D or 3D images. This technique can yield very high-resolution images, with synchrotrons having the capacity to produce images with resolutions in the nanometer range. Neutron radiography and tomography rely upon similar principles as X-ray radio/tomography, producing images through the attenuation of neutron beams passing through the sample instead of X-rays. As neutrons interact with nuclei and x-rays with electrons, these two methods produce different images. Air-coupled ultrasound produces non-invasive structural images via the analysis of ultrasonic wave propagation through the sample, allowing the observation of thickness resonances. Air-coupled sensors allow this method to be performed without coming into contact with the sample [22]. Laser-based methods using guided waves can give information on structure by studying the propagation of pulsed laser-induced waves within the sample [23]. The mechanical properties of plants that can be elucidated via both guided wave techniques and air-coupled ultrasound can be linked to plant water status. Finally, 2D light transmission can be used to generate images of root structure based upon the attenuation of visible light through a translucent soil medium as areas of higher water content will transmit more light.

Xylem flow can be measured in several ways. Dye tracing [27] can be used to measure xylem flow velocity by observing the time taken for dye to cross two points on a trajectory. By irrigating plants using water labeled with deuterium, xylem velocity and dynamic water distribution can be measured. Information on xylem flow can also be obtained with sap flow meters that are placed around stems and which function using a variety of heat pulse-based methods. Non-invasive methods exist such as gravimetric analysis which consist of following the evolution of the weight of potted plants to measure the loss of water by transpiration, considering the evaporation of the soil by covering it. Porometers can non-destructively provide indirect estimates of whole plant transpiration by measuring stomatal conductance at the level of the leaf.

Established and reliable methods to measure phloem flow are less numerous. ^11^C PET imaging can be used to follow the flow of radiotracers in order to obtain information about phloem [28, 29]. This technique extrapolates flow information by measuring the time it takes for radiotracers to pass two points along a pathway. Phloem flow rate can also be estimated by exploiting the ability of aphids to penetrate phloem tissues in certain plant organs [30]. By using radiolabeled CO_2_ and placing several aphid stylets along a plant organ, one can extrapolate pressure differences which can be used to calculate phloem flow rate.

There are a couple of methods used to measure cavitation uniquely. One of these methods, developed in our laboratory (PIAF, INRAE), uses a xylem embolism meter, the XYL’EM device, to study the relationship between xylem conductance and cavitation [31]. The level of cavitation of a branch can be estimated by measuring the conductivity of water moving through the branch just after being cut, and then on the same branch at full saturation after removing the air bubbles. There are methods using the production of acoustic emissions to measure cavitation. These rely on detecting sounds in the ultrasonic frequency range which are produced by the plant during dehydration and relating these sounds to the hydraulic conductivity of the plant [32].

### Measuring water content and imaging plant structure with portable MRI

**Fig. 2:**
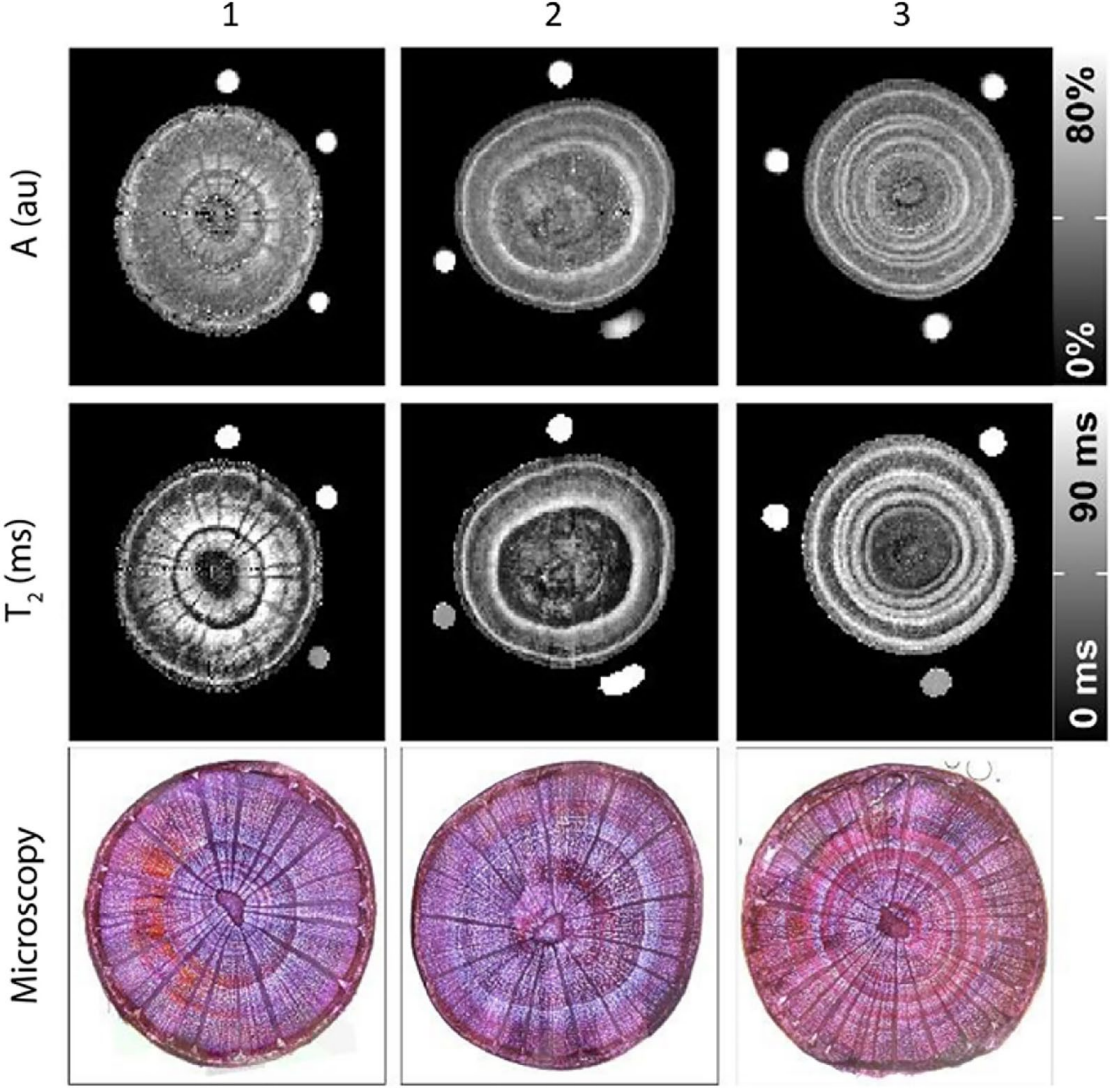
Proton density (A) and transversal relaxation time (*T*_2_) maps acquired with a 0.25 T-MRI, and microscopy images of the stem of three *Fagus sylvatica* samples (1–3). The proton density is related to water content (%) (Adapted from [10] with permission from Wiley)

MRI techniques are not only non-invasive and direct, but they are versatile. For example, the quantity of water within each voxel can be measured by using quantitative MRI, and information about plant structure can be obtained using relaxation-weighted MRI. This is possible because, as previously evoked, the MRI signal is directly related to water content, and relaxation times give information on the environment of water molecules. For instance, using relaxation times, *T*_2_-weighted maps make it possible to observe different tissue compartments, at the intracellular or intratissue scale. This has been demonstrated in the plant sciences using portable MRI devices, primarily on deciduous tree branches [10, 33, 34] and fruits [35]. Growth rings, xylem, and phloem distribution have been successfully imaged. Jones et al*.* developed a portable MRI system called the Tree Hugger which successfully imaged tree stems, and was sensitive enough to capture both diurnal and seasonal variation [36]. This device also managed to capture variation of water content within the trunk, putting in evidence “grain rotation” where water flows through the trunk in a spiral. It has also been demonstrated that portable MRI is capable of measuring the water content of leaves, as well as studying how leaf water content evolves diurnally and in the context of hydric stress [37]. At the level of underground plant organs, the difference in relaxation times between the soil and the roots permitted the direct imaging of roots within intact soil and to observe their distribution [13, 38]. Figure 2 shows how proton density measurements and *T*_2_ relaxation times can be used to create weighted images, and these images are comparable to what can be obtained with microscopy.

### Measuring sap flow and physiological processes with portable MRI

#### Sap flow

**Fig. 3:**
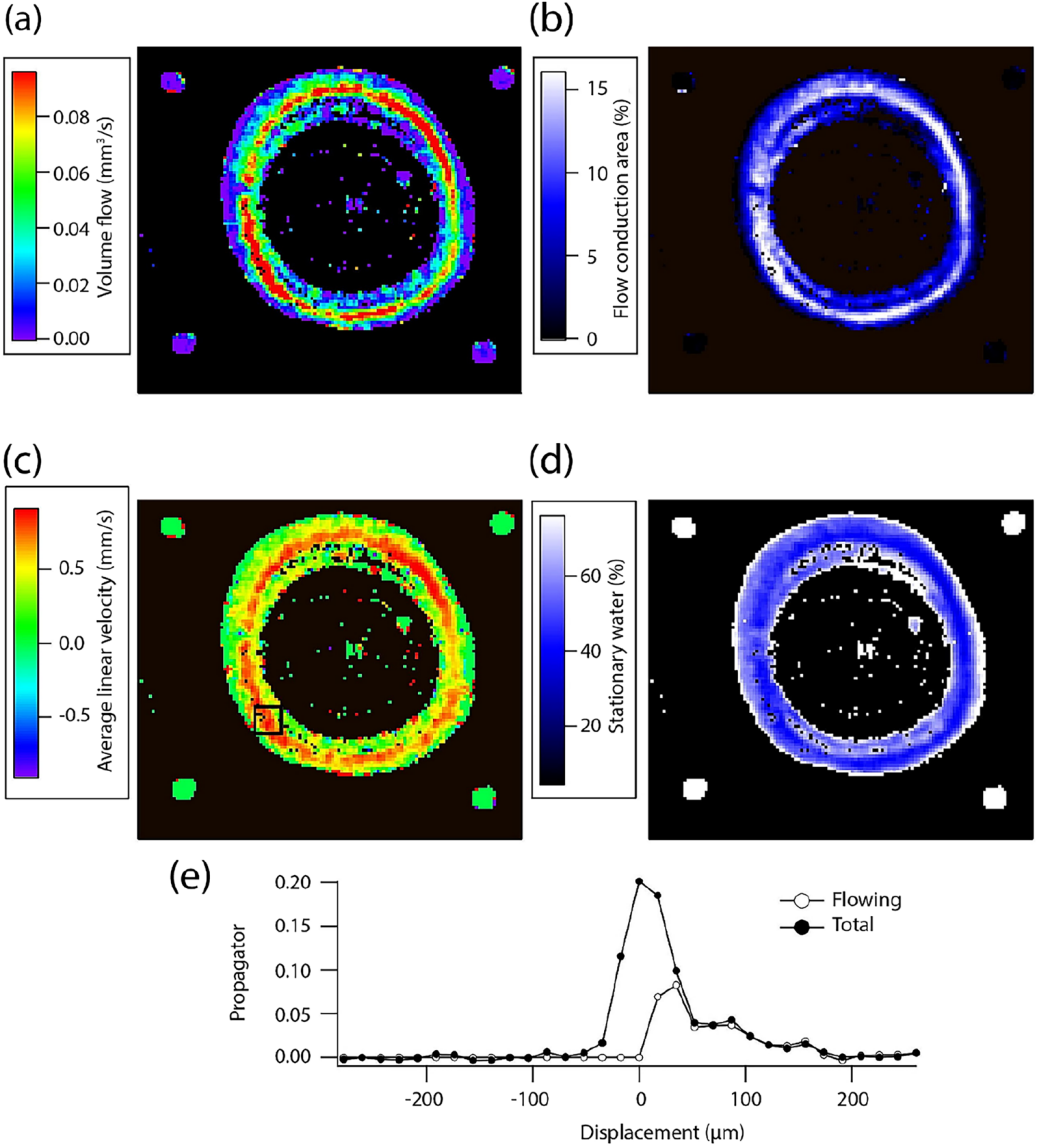
Flow parameters: volume flow (**a**), flow conducting area (**b**), average linear velocity (**c**), and amount of stationary water (**d**). The propagator displayed (**e**) was measured in the pixel represented by a black square in (**c**). These measurements were acquired with a 0.2 T-MRI on a Zelkova tree in situ (Adapted from [33] with permission from Elsevier)

Both laboratory and portable MRI are exceptionally interesting tools to measure sap flow because they enable the measurement of both the xylem and phloem fluxes by yielding a probability of flow rate, called a propagator, as seen in Fig. 3e. This is acquired by spatially labelling water molecules and then measuring their displacement per unit of time. Laboratory MRI velocimetry has been used to measure xylem flow for decades, in addition to phloem flow, with the limitation that it cannot be taken into the field [6, 39]. The feasibility of using portable or potentially portable devices has already been demonstrated. Van As et al. performed the first measurements on plants with a portable MRI device in 1994 and demonstrated that flow and *T*_2_ relaxation times varied with light intensity [40]. Also using portable devices, Windt et al*.* measured the xylem and phloem fluxes in the stems of four different plant species: poplar trees, tomato, tobacco and castor bean [12]. They were able to observe that the speed, i.e., the average velocity, of the phloem was not very substantial compared to the xylem, regardless of species, measured between 0.25 and 0.40 mm s^−1^, while that of the xylem was higher and more differentiated, from 1.6 mm s^−1^ for poplar to 5.10 mm s^−1^ for tomato. They also noticed a variation of the fluxes according to the diurnal cycle, the average speed of the fluxes decreasing at night regardless of the vessel studied. Nagata et al*.* measured xylem flow in a tree (Zelkova) outside the laboratory [33]. They showed this same day/night cycle in connection with measurements of sap flow carried out elsewhere, and an evolution of these fluxes according to the season, the latter decreasing in autumn and winter due to the loss of leaves. Figure 3 presents an example of how sap flow and its distribution can be measured over an entire plant organ.

#### Physiological processes

Non-invasive imaging methods permit the study of physiological processes. Root water uptake has been studied by X-ray [41, 42] and neutron tomography [21, 43–45] and also by 2D-light transmission imaging [24, 25]. These methods enable a spatially resolved study of root water uptake. Recently, Bagnall et al*.* demonstrated with a low-field MRI device that roots can be detected and imaged in natural soils, helping to better understand root morphology, architecture, and development [13, 38]. But, as far as we know, spatially resolved root water uptake has not yet been studied with portable MRI. Nonetheless, diurnal variations of proton density and relaxation times have been observed in the roots of herbaceous species [46]. Such variations in proton density were also observed in the stems of trees [34, 47]. In the cited studies, MRI parameters, proton density and *T*_2_, were elevated at the end of the night and decreased during the day. This evolution was consistent with the physiology of the plant which transpired during the day.

**Fig. 4.**
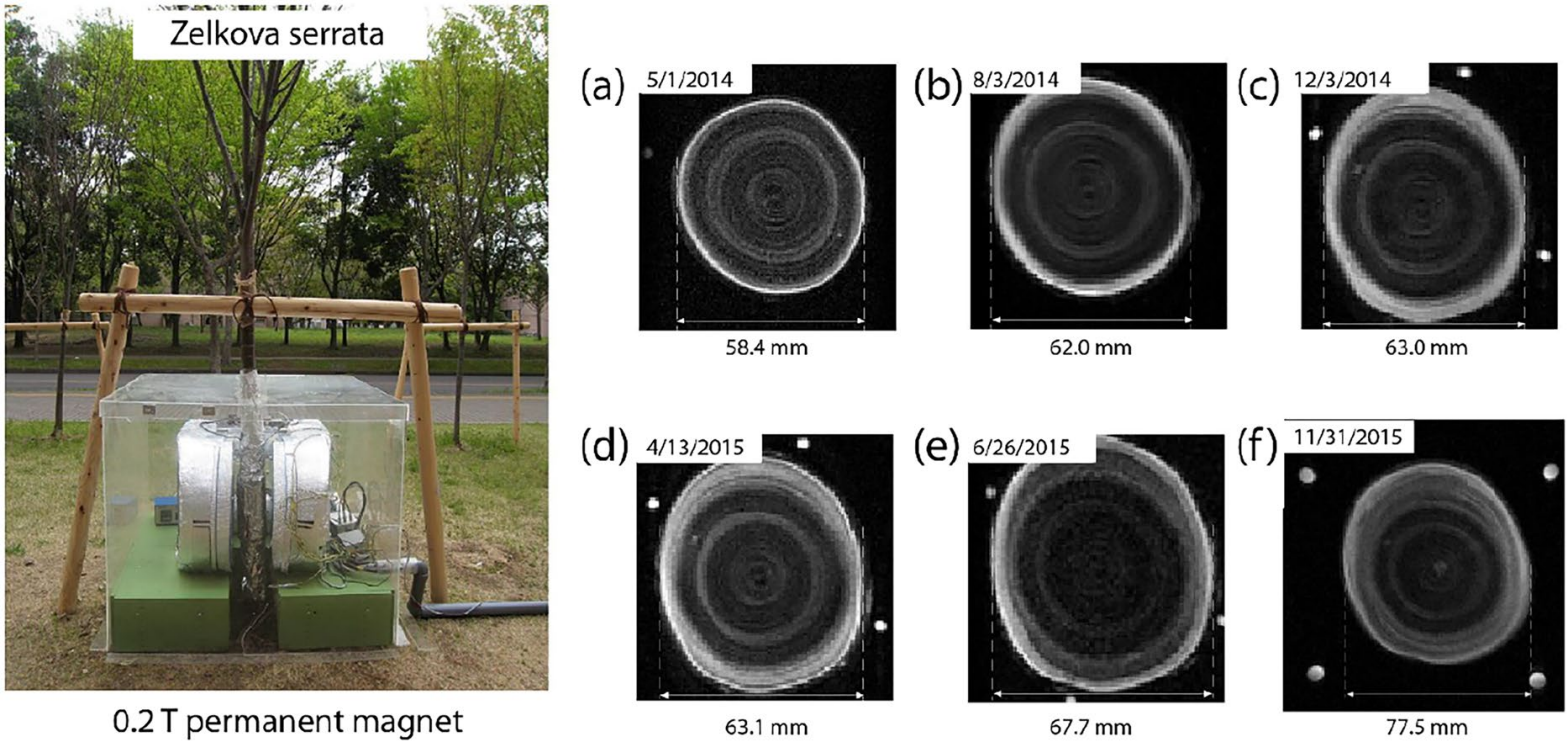
0.2 T-MRI positioned outside the laboratory on the stem of *Zelkova serrata*. Images (**a**–**f**) shows the growth of the stem, an increase of the stem diameter being observed (Adapted from [33] with permission from Elsevier)

Organ growth has also been successfully studied using portable MRI. Windt et al*.* showed that there was a change in the amount of water in trees at night, with an observation of the growth of the trunk after a few days [47]. At night, the quantity of water in the tree increased, then a portion was used at the beginning of the day, followed by a stabilization in the amount of water present in the trunk. As the days passed, the overall amount of water present in the trunk increased, reflecting growth of the trunk. Similar results were observed in oak with an increase in water uptake at night. A link between the diameter of the stem and the amount of water absorbed has also been established, the latter being greater when the diameter of the stem increases [48]. While these studies were conducted in a climate chamber, Nagata et al*.* followed the growth of a tree outside the laboratory, in the plant’s natural environment [33]. They observed that tree growth was rapid during the spring and summer months via the visualization of growth rings. Growth slowed after the summer months, and stopped completely during the winter months, until the following summer with a slight increase in trunk diameter (Fig. 4).

While physiological processes, such as growth, have been studied with direct measurements (visualization of growth rings or variation of the amount of water), they can also be studied indirectly with the evolution of relaxation times which can be related to the evolution of the structure and/or function of organs. Using a portable device, growth and senescence processes have been studied on leaves using relaxation times. Musse et al*.* demonstrated that three or four *T*_2_ relaxation times were present depending upon leaf phenology. These relaxation times were attributed to cellular compartments such as the vacuole, the cell wall or even the dry matter. While the shorter relaxation times disappeared with increasing levels of development, the longer relaxation times increased in proportion. This was due to the increase in the size of the vacuole [49]. In senescing leaves, the longest *T*_2_ relaxation time split into two components. This has been attributed to tissue heterogeneity, namely, palisade cells [50, 51]. Using portable devices, relaxation times and other NMR parameters have also been used to study the growth of fruits. Geya et al*.* showed an increase in the value of relaxation times and ADC in conjunction with increasing fruit weight [52]. Another study followed the maturation of tomatoes and showed that three structures composing the fruit were visible at the beginning of the experiment (pericarp, loculus and placenta), while the pericarp and loculus were no longer distinguishable at the end of the experiment on mature fruit. The level of ripening could also be quantified due to the pericarp/locule ratio which increased with the maturity of the fruit [35].

### Measuring stress and disease impacts with portable MRI

**Fig. 5:**
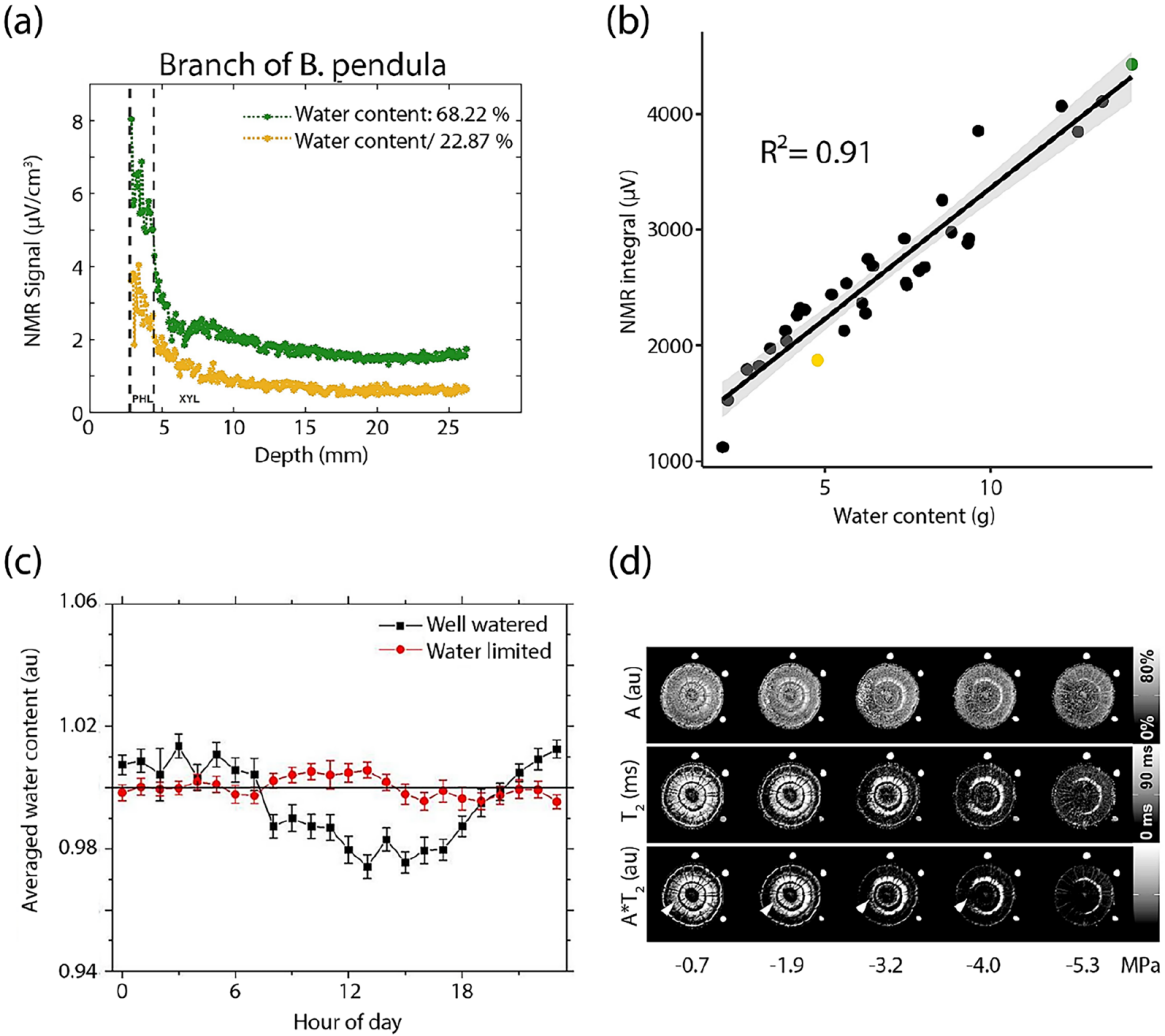
Profiles acquired on (**a**) the branch of a birch tree, *B. pendula* (Blystone et al., unpublished). The peak between the dashed lines corresponds to the bark zone, including the phloem tissue (PHL), while the broader peak at greater depths corresponds to xylem tissue (XYL); (**b**) Linear regression of the integral of the NMR signal as a function of water content, taken through time, on branches of silver birch trees. The green and yellow points correspond to the profiles of subset a. (**c**) Diurnal evolution of the average water content in an Aspen tree under well-watered (black) and water limited conditions (Adapted from [56] with permission from Frontiers). (**d**) Quantitative maps of a *Fagus sylvatica* stem according to the water potential (Adapted from [10] with permission from Wiley)

Portable MRI enables the study of the impact of hydric stress and the monitoring of water dynamics in plants submitted to drought conditions, while overcoming the challenges of alternative methods. Scheenen et al. induced embolism in the stem of a cucumber plant by cooling the roots, and used a potentially portable MRI device to observe the formation and recovery of embolized vessels by taking flow measurements [53]. Using a portable and unilateral device, other authors (Blystone et al*.,* unpublished) were able to differentiate the xylem and phloem tissues of birch tree branches by taking profiles, the signal intensity in function of measurement depth, as seen in Fig. 5A., as well as observe a linear correlation between the water content of the branches and the integral of the profiles as the branches dehydrated. These authors were also able to observe how the distribution of water in the xylem and phloem zones changed throughout the dehydration process. Besides birch tree branches, similar results were observed in leaves, stems, branches and roots of other herbaceous plants and trees [10, 37, 54–57]. Contrary to hydrated plants, several studies found no observable nychthemeral variations in the NMR signal in the presence of water stress [55–57]. Peuke et al. captured variations in both xylem and phloem fluxes in potted castor bean plants according to anoxic treatments in both roots and shoots, and put in evidence a loss of circadian variation in the xylem flux after anoxia in the root zone. All of these results were obtained based on semi-quantitative data. However, drought impact can be studied with transverse relaxation times. A decrease in mean *T*_2_ has been demonstrated in leaves, branches and roots submitted to water stress [10, 54, 57, 58]. Portable MRI has also been used to study embolism formation and spreading. It has been shown in studies on tree organs that embolism begins in the oldest xylem vessels before spreading to the youngest [10, 59]. A good correlation has been observed between the areas of xylem filled with water and the areas with the highest signal on the MRI, and between the curves of vulnerability and the embolized area, giving the possibility of estimating the areas of xylem impelling water movement [59].

Besides studying drought impact, portable MRI has also been used to study the influence of diseases on anatomy and water dynamics. In 2011, Japanese researchers developed a portable MRI device to characterize the physiological differences between the branch of a healthy pear tree and the branch of a pear tree affected by dwarfism [11]. These authors observed a difference in relaxation times, ADC values, and their distributions, between the diseased and healthy branches, which translated to the visualization of the yearly rings in healthy trees, and the absence in diseased ones. Using a portable device, Umebayashi et al. followed the progression of pine wilt disease and its impact upon xylem embolism by imaging water distribution over time [60]. These authors used *T*_1_ weighted conditions to show that embolism spread in two different ways, both in a growing number of small patches and the spreading of a larger patch at the site of nematode inoculation. Utsuzawa et al. also followed the progression of pine wilt disease using a potentially portable MRI device and *T*_1_-weighted conditions [61]. They were able to show that the area and speed of cavitation spread in two stages: a first stage characterized by gradual and confined propagation, and a second stage characterized by rapid propagation until it occupied most of the xylem tissue.

Table 1 summarizes all the references mentioned in this review concerning the use of portable or potentially portable MRI to measure plant structure or physiology.

**Table 1:** A non-exhaustive tour of the literature of portable or potentially portable MRI applied to plants

Organ	Reference	Species	B_0_ (T)	Highlights
Fruit	Baek et al*.* [35]	*Solanum lycopersicum* cv. Tiara, Tiara TY, and Unicorn	1	Variation in signal intensity as a function of maturity; Assignment of maturity based on the pericarp/locule signal ratio
	Geya et al*.* [52]	*Pyrus pyrifolia*	0.2	Variation in *T*_2_ as a function of fruit growth and changes in tissue structure; Linear relationship between relaxation rates and the inverse of the cube root of pear fruit weight
	Windt and Blümler [48]	*Phaseolus vulgaris* L	0.26	Monitoring of fruit water content and fruit growth
Leaves	Capitani et al*.* [54]	*Zea mays*; *Phaseolus vulgaris*; *Populus nigra*; *Cistus incanus*; *Quercus ilex*	0.4	Relationship between the integral of the NMR signal and leaf water status, and with the rate of transpiration in hydrated leaves; Reduction in NMR signal with leaf dehydration; Different trends between species depending on their water strategies
	Musse et al*.* [49]	*Brassica napus* L., genotype Tenor	0.47	Access to cell structure by measuring *T*_2_ through subcellular water distribution; Variation in *T*_2_ distribution as a function of leaf developmental stage
	Musse et al*.* [51]	*Brassica napus* L., genotype Aviso	0.47	Reflection of the senescence process in the variation of *T*_2_ distribution; Earlier change in *T*_2_ than in chlorophyll and dry matter content
	Sorin et al*.* [50]	*Brassica napus* L., genotype Tenor	0.47	Access to cell structure by measuring *T*_2_ through subcellular water distribution; Variation in *T*_2_ distribution as a function of leaf developmental stage
	Sorin et al*.* [58]	*Nicotiana tabacum* cv. Xanthi	0.47	The stage of leaf development mainly affects the longest *T*_2_; The impact of water stress depends on the plant's stage of development and the intensity of the stress
	Windt et al*.* [37]	*Oryza sativa* cv. Nuovo Maratelli	0.242	Diurnal variation in leaf water content; Weaker tendency in case of osmotic stress
Stem	Fukuda et al*.* [59]	*Cercidiphyllum japonicum*; *Betula platyphylla* var. japonica	1	Measurement of water content; Visualization of emboli propagation; Estimation of the relative area of the embolism from MR images in accordance with vulnerability curves
	Homan et al*.* [39]	*Prunus*	0.7	Similar flow measurements at high and low magnetic fields
	Jones et al*.* [36]	*Prunus padus*	0.025	Imaging water distribution; Correlation between signal intensity and meteorological conditions; Diurnal and seasonal variation in water status
	Kimura et al*.* [11]	*Pyrus pyrifolia*	0.3	Clear difference between the ADC map of the branch affected by dwarf disease and the ADC map of the normal branch; Variation in microscopic water flow in the branch as a function of solar radiation
	Malone et al*.* [56]	*Populus tremuloides*; *Juniperus monosperma*; *Pinus edulis*	9E-04	Diurnal variation of the NMR signal in a well-watered plant; No clear diurnal variation of the NMR signal in a water-limited plant
	Meixner et al*.* [10]	*Fagus sylvatica*	0.25	Measurement of water content; Visualization of emboli propagation;
	Meixner et al*.* [34]	*Malus domestica* cv. Captan; *Fagus sylvatica*	0.25	Imaging water distribution; Diurnal variation in water content and *T*_2_
	Nagata et al*.* [33]	*Zelkova serrata*	0.2	Monitoring tree growth; Diurnal and seasonal variation in xylem flow
	Peuke et al*.* [62]	*Ricinus communis* L	0.72	Reduction in phloem and xylem flows during root anoxia treatment; No diurnal variation in xylem sap flow after anoxia treatment; Reduction then recovery of phloem sap flow during shoot anoxia treatment
	Scheenen et al*.* [53]	*Cucumis sativus* cv. Hurona	0.7	Reduction in root water uptake and xylem hydraulic conductance due to emboli formation after cold stress; Refilling monitoring
	Umebayashi et al*.* [60]	*Pinus thunbergii*	0.3	Imaging the spread of embolism in pine wilted disease caused by nematodes; Characterization of two types of embolism, one of which spread in all directions and was caused by the nematode population staying around the inoculation site
	Utsuzawa et al*.* [61]	*Pinus thunbergii*	1	Visualisation of xylem cavitation and the spread of embolism caused by nematodes; Area and speed of cavitation spreading in two stages: a first one with gradual and confined propagation, and second, rapid propagation progressing until it occupied most of the xylem
	Van As et al*.* [40]	*Cucumis sativus* L	0.235	Variation in flux and *T*_2_ with light intensity
	Windt et al*.* [12]	*Populus tremula* × *Populus alba*, INRA clone 717 1B4; *Ricinus communis*; *Lycopersicon esculentum* cv. Counter; *Nicotiana tabacum* cv. Petit Havana SR1	0.72	Measurement of phloem and xylem flow; Diurnal variation in xylem flow
	Windt et al*.* [47]	*Ricinus communis*; *Populus nigra*	0.57	Non spatially resolved xylem flow measurement; Diurnal variation in water status; Monitoring the growth of a tree trunk
	Windt and Blümler [48]	*Populus nigra* L.; *Quercus robur* L	0.57 0.26	Measurement of xylem flow; Monitoring water content, stem diameter growth and shrinkage
	Yoder et al*.* [55]	*Populus tremuloides*; *Pinus edulis*; *Juniperus monosperma*; *Pinus ponderosa*	9E-04	Decrease in signal amplitude as the tree dries out
Roots	Bagnall et al*.* [38]	*Sorghum bicolor* (L. Moench)	0.047	Measuring soil and root *T*_2_; Soil *T*_2_ variation as a function of soil type; Imaging roots in the soil
	Bagnall et al*.* [13]	*Sorghum bicolor* (L. Moench)	0.047	2D and 3D imaging of root architecture in soils; Root segmentation
	Nuixe et al*.* [46]	*Dactylis glomerata*; *Plantago lanceolata*; *Medicago sativa*	0.3	Diurnal variation in water content and in *T*_2_

### Comparison of portable MRI and alternative methods

Portable MRI is capable of measuring water content directly, non-destructively, quantitatively, and in the field. Alternative methods to measure water content, such as taking fresh and dry weights or using NIRS, come with certain disadvantages. Taking fresh and dry weights has the advantage of being direct and quantitative, but it is forcibly destructive. NIRS enables water content measurements non-destructively and in situ, but it is indirect as the method relies upon calibration models which need validation for each species tested in order to interpret spectra. The interpretation of spectra can be difficult due to the fact that a variety of spectral information on pigment uptake, diffusion and leaf structure can obscure water information [63]. Analysis with NIRS is also limited to the outermost layers of a sample. THz spectroscopy, while relatively inexpensive and a non-ionizing alternative to X-ray-based imaging, has the disadvantage of being indirect, as models must be used to link data to plant water content. Modeling becomes further complicated in the context of in situ measurements, and a large number of plants would have to be measured in order to render useful models. Relying upon models presents a challenge with regard to the universal application of a method, as models are often specific to a certain plant species, or even to specific experimental conditions. Like NIRS, the THz method is also limited to the superficial layers of the sample.

Regarding methods to study plant structure, microscopy techniques, 2D-light transmission imaging, X-ray and neutron radiography/tomography are all limited to laboratory use. Portable MRI is able to transcend the challenge of taking structural measurements in the field. In addition, microscopy techniques are destructive [64], as are dye tracing techniques. Due to ionizing radiation, X-ray radio/tomography can also impact natural processes within the plant [65]. 2D-light transmission imaging is especially limited as it cannot be used with an opaque medium [66] such as the soil found in a plant’s natural environment. It also requires the use of a very thin rhizotron which limits the plant species that can be studied. Portable MRI techniques are capable of overcoming these challenges. Air-coupled ultrasound is non-invasive, non-destructive, and has a high temporal resolution. However, this technique is indirect and relies upon the interpretation of ultrasonic parameters, which can be impacted by multiple variables inherent to the sample, creating results with large variation [22]. The use of laser-based guided waves equally yields results with variation as the spectral response is impacted by biological processes that vary spatially and temporally. Both air-coupled ultrasound and guided wave techniques are indirect as information about plant water status is extrapolated from mechanical information yielded by the measurements. Moreover, the laser beam used for guided waves techniques can also cause tissue damage [23].

MRI techniques permit the imaging of cavitation and following the dehydration process of a plant by directly imaging water content locally. Alternative methods to measure cavitation, such as the XY’LEM system and methods based on acoustic emissions, are limited in scope and application. The XY’LEM system is forcibly destructive, it must be carried out in the laboratory, and it only gives information on one branch. Moreover, the system links the conductivity over the entire branch with cavitation percentages, giving no indication of local dehydration. Methods based on acoustic emissions are limited in that they are more qualitative than quantitative. Moreover, linking acoustic emissions to the amount of cavitation in a sample relies upon the assumption that the plant vulnerability curve has a perfect sigmoid shape, and also that the end point, where there is 100% cavitation, is known [32], providing results with a large margin of error.

One of the major advantages of MRI techniques is that they provide a multitude of information; one can measure the water content and the flow of water with the same device. Alternative methods to measure sap flow give information only on flow, as well as presenting other disadvantages. While dye tracing is inexpensive and easy to implement, it is destructive as plant organs must be sampled to measure dye accumulation. Using deuterium to measure flow can be done in situ, but the method is invasive and relies upon models to interpret the results, and relying upon models leads to limitations previously evoked [67]. Sap flow meters can also be used in situ, however, they are equally invasive [68]. Gravimetric methods require the plant to be in a container that can be placed on a scale, eliminating the possibility of monitoring a plant in its natural environment. While porometers can be used outdoors in a plant's natural environment, the estimation of plant transpiration comes with a large margin of error due to variation between leaves and location within the canopy. Beyond MRI, methods to measure phloem flow are even more limited. PET imaging is non-invasive and direct, but its use is limited due to the necessity of expensive and cumbersome laboratory equipment which is needed to generate radiotracers [28, 29]. While using aphids to measure phloem flow does not require expensive equipment, the technique is limited to plant species with a corresponding insect host, and to tissues in which the aphid can penetrate. It is also difficult to accurately measure the rate of phloem transport because the phloem quantities are in the nanoliter range and evaporation has a significant impact on the measurements [30].

Portable MRI, however, does present its own unique challenges. In comparison to most of the alternative methods mentioned, portable MRI devices are often made in-house. This means that there are very few commercialized devices available with standard protocols, and this limits the widespread use of the method. Moreover, the smaller magnet size of portable devices leads to a loss of resolution which can limit the use of portable MRI with regard to imaging certain microstructures, such as the cambium cell layer in trees. Magnets are also sensitive to temperature, which can create complications with an apparatus that is used outdoors in highly variable conditions. The shape and orientation of devices can also limit their application to plants. Devices with enclosed magnetic fields can limit the size and shape of organs that can be measured, and horizontally oriented devices are limited in the field where organs, such as stems, have vertical orientations. Finally, the manipulation of experimental MRI parameters and the interpretation of results often demands specialized knowledge in the field of MRI, making magnetic resonance techniques difficult to apply for the non-expert user. Nonetheless, these drawbacks are compensated by the ability of portable MRI to non-invasively study plant structure, physiology, and plant water dynamics outside of the laboratory, and using only one tool.

The advantages and disadvantages of each method discussed in this review, alternative to portable MRI, are summarized in Table 2. We defined a method as not easily accessible when it requires constraining laboratory equipment, such as specific lead shields, as is the case with the use of X-ray imagers, or when the access for plant work is difficult (geometry and orientation of the device). We considered a method as easy to perform when the execution of the experiment requires few steps. We consider a method to be affordable if it is significantly cheaper than the majority of portable MRI devices.

**Table 2:** Advantages and inconveniences of methods, alternative to portable MRI, used to measure water content, plant structure, xylem and phloem fluxes, and cavitation

Parameter measured	Method	Advantages	Disadvantages
Anatomy	Microscopy	Affordable; easy to perform; high spatial resolution (from a few hundredths of a nanometer for electronic to a couple of tenths of a micrometer for optic)	Destructive; Limited to laboratory; only a small portion of sample can be observed at once
	X-ray (µ)radiography	High spatial (~ 50–100 μm) and temporal (~ tens of seconds per scan) resolution; non-invasive	Limited to laboratory; possible impact of ionizing radiation; expensive; not easily accessible
	Neutron radiography	High spatial (~ 100 μm) and temporal (~ tens of seconds per scan) resolution; non-invasive; adapted to dense materials	Limited to laboratory; expensive; not easily accessible; possible impact of neutron radiation on plant tissue; limited to plants that fit in a thin container
	Dye tracing	Affordable; easy to perform; portable	Destructive
	Air-coupled ultrasound	Non-invasive; high temporal resolution (a few seconds per scan); easy to perform; affordable; portable	Indirect; large variation in results
	Laser-based guided waves	High temporal resolution (~ a few seconds per scan); easy to perform; portable	Indirect; large variation in results; laser beam can cause tissue damage
	High-field MRI	Non-invasive; high spatial (~ 100 μm) and temporal (from a few minutes to a few hours depending upon the experiment) resolution	Limited to laboratory; expensive equipment and maintenance; not easily accessible; mainly horizontal orientation; limited plant size and shape
Water content	Fresh weight/Dry weight	Affordable; Easy to perform	Limited to laboratory; Destructive; time consuming (overnight drying required)
	NIRS	Non-invasive; portable; affordable; easy to perform	Indirect, results must be interpreted through models (problem of genericity); calibration particularities for a given species and/or context; limited to outermost layers of sample
	THz spectroscopy	Non-invasive; easy to perform; affordable; portable	Indirect; results must be interpreted through models; limited to outermost layers of sample
	High-field MRI	Non-invasive; high spatial (~ 100 μm) and temporal (from a few minutes to a few hours depending upon the experiment) resolution	Limited to laboratory; expensive equipment and maintenance; not easily accessible; mainly horizontal orientation; limited plant size and shape
Xylem flux	Sap flow meters	Portable; affordable; easy to perform	Invasive; limited to organs with sufficient diameter
	Porometers	Portable; affordable; easy to perform	Indirect, large margin of error in estimating sap flow due to variation between leaves
	2D light transmission	Non-invasive; affordable; spatial resolution (~ 200–500 μm), offers real-time data on water uptake by roots	Indirect, limited to laboratory; limited to certain plants that fit in a thin container and sandy soil; no other light sources than the instrument during measurement
	Gravimetric	Affordable; portable; easy to perform; non-invasive	Indirect
	Isotopic tracing	Portable	Destructive; results must be interpreted through models; can be expensive (cost of radiotracers and mass spectroscopy analyses)
	Dye tracing	Affordable; easy to perform; portable	Destructive
	High-field MRI	Non-invasive; high spatial (~ 100 μm) and temporal (from a few minutes to a few hours depending upon the experiment) resolution	Limited to laboratory; expensive equipment and maintenance; not easily accessible; mainly horizontal orientation; limited plant size and shape
Phloem flux	Aphids	Affordable	Limited to laboratory; Indirect, limited to certain plant species; approximative with large margin of error; can be complicated to execute
	^11^C PET imaging	Non-invasive; sufficient spatial resolution to capture water dynamics (a couple of millimeters) and high temporal (a few minutes) resolution	Limited to laboratory; expensive and cumbersome equipment; possible impact of ionizing radiation
	High-field MRI	Non-invasive, high spatial (~ 100 μm) and temporal (from a few minutes to a few hours depending upon the experiment) resolution	Limited to laboratory; expensive equipment and maintenance; not easily accessible; mainly horizontal orientation; limited plant size and shape
Cavitation	Acoustic emissions	Portable; easy to perform; affordable	Indirect, more qualitative than quantitative, can be destructive
	Dye tracing	Affordable; easy to perform; portable	Destructive
	Xylem Embolism Meter	Affordable; easy to perform	Indirect, Destructive; limited to laboratory; not suitable for very small (fine roots) or large samples (> 10 cm diameter)
	Microscopy	Affordable; easy to perform; high spatial resolution (from a few hundredths of a nanometer for electronic to a couple of tenths of a micrometer for optic)	Destructive; Limited to laboratory; only a small portion of sample can be observed at once
	X-ray (µ)radiography	High spatial (~ 50–100 μm) and temporal (~ tens of seconds per scan) resolution; non-invasive	Limited to laboratory; possible impact of ionizing radiation; expensive; not easily accessible
	Neutron radiography	High spatial (~ 100 μm) and temporal (~ tens of seconds per scan) resolution; non-invasive; adapted to dense materials	Limited to laboratory; expensive; not easily accessible; possible impact of neutron radiation on plant tissue; limited to plants that fit in a thin container
	High-field MRI	Non-invasive; high spatial (~ 100 μm) and temporal (from a few minutes to a few hours depending upon the experiment) resolution	Limited to laboratory; expensive equipment and maintenance; not easily accessible; mainly horizontal orientation; limited plant size and shape

## Conclusion

Portable MRI is an extremely useful tool in the plant sciences and presents a multitude of advantages over alternative methods. It has mainly been used on trees, but applications can be found on other types of plants, such as herbaceous or crop species. By studying water in a plant, portable MRI is capable of measuring plant structure, physiology, stress response, disease, water content, and flow, all non-invasively, directly, and in the field. Thus, portable MRI can extract a multitude of information using one method, whereas multiple alternative laboratory methods would otherwise have to be used in combination. While there is still work to be done to increase its accessibility to plant biologists, portable MRI stands as a promising method to study plant structure and functioning.

## Data Availability

Data sharing is not applicable to this article as no datasets were generated or analyzed during the current study.

## Abbreviations

ADC: Apparent diffusion coefficient
B_0_: Principal magnetic field
CO_2_: Carbon dioxide
MRI: Magnetic resonance imaging
NIRS: Near infrared spectroscopy
NMR: Nuclear magnetic resonance
RWC: Relative water content
SNR: Signal-to-noise ratio
*T*_1_: Longitudinal relaxation time
*T*_2_: Transverse relaxation time
